# The Design of SPRINT MIND and SPRINT MIND 2020

**DOI:** 10.1002/alz.095412

**Published:** 2025-01-09

**Authors:** Stephen R. Rapp, Mark A. Supiano

**Affiliations:** ^1^ Wake Forest University School of Medicine, Winston‐Salem, NC USA; ^2^ University of Utah Health, Salt Lake City, UT USA

## Abstract

**Background:**

The Systolic Blood Pressure Intervention Trial (SPRINT) included a specific aim on the effect of blood pressure lowering the risk of cognitive impairment (mild cognitive impairment (MCI) and dementia). Early termination of the trial and fewer than expected cases left the study under powered to detect cases of probable dementia, but the intervention reduced the risk for MCI and for a composite of MCI or probable dementia (PD). To increase the length of follow‐up and number of cases of probable dementia, two studies were funded: one from October 2017 through June 2018 and a second, the SPRINT MIND 2020 study, from December 2019 through December 2023.

**Method:**

In the main trial, 9361 participants were randomized to either Intensive or Standard BP control, systolic BP < 120 or < 140 mmHg, respectively. For the cognitive aim, outcomes were adjudicated PD and MCI.

**Result:**

Of the 9361 participants in the SPRINT trial, 8563 completed one or more cognitive assessment, and 325 were adjudicated to have probable dementia at the end of the first extended follow‐up. The SPRINT MIND 2020 follow‐up ascertained an additional 216 cases of probable dementia. An additional 170 cases of mild cognitive impairment were also ascertained.

**Conclusion:**

SPRINT MIND 2020 successfully extended follow‐up time for the SPRINT MIND study and increased the number of observed cognitive outcomes substantially enabling an analysis of legacy treatment effects.

